# The Lutheran Imaginary That Underpins Social Democracy

**DOI:** 10.3389/fpsyg.2021.746406

**Published:** 2021-09-10

**Authors:** Mads Larsen

**Affiliations:** European Languages and Transcultural Studies, The University of California, Los Angeles, Los Angeles, CA, United States

**Keywords:** cultural psychology, social democracy, fission-fusion, evolutionary literary criticism, psychological-institutional coevolution, socialism, Lutheranism, statist individualism

## Abstract

Scandinavian social democracy is increasingly upheld as an alternative that could reform capitalism. The Nordic Model produces income equality, low-conflict politics, and happy people. When half of young Americans express that they would prefer “socialism,” they generally mean to live in a society that provides for its citizens as the Nordics do. Such aspirations are complicated by how social democracy can be viewed as a secularized form of Lutheranism, the Protestant creed that the Nordic region embraced in the 16th century. Lutheran norms and values carried into the modern era and made possible social democracy's two distinguishing features: fascist corporatism and socialist redistribution. A strong state facilitates *statist individualism*, which empowers individuals vis-à-vis employers, parents, and spouses. The outcome could be cross-culturally salient, as it brings people closer to our species' fission-fusion baseline. Yet in the modern environment, only Nordics seem to have a cultural imaginary that makes compelling the politics that drive such high levels of both productivity and egalitarianism. The region's storytelling reflects this Lutheran past and is used to negotiate modern adaptations. A better understanding of social democracy could help prevent that demands for “socialism” motivate a turn to actual socialism.

## Introduction

Henrich (2020) accounts for how modernity arose from the psychological-institutional coevolution set in motion by the Church's dissolution of kinship societies. Freed from kin bonds, the Western individual evolved toward ever greater independence. Liberal humanism, with its sanctification of the individual, became the imaginary that underpinned the modern world. The liberal utopia promised that granting freedoms to individuals would create a global society of peace and prosperity. Free markets would reduce between- and within-nation inequalities, thus reducing the potential for conflict. In the 21st century, these predictions at best appear naïve. As the liberal utopia played itself out—like the fascist and socialist utopias did in the past century (Harari, 2014)—Scandinavian social democracy caught the attention of progressives. From a liberal perspective, the Nordics' large-government, high-taxation model restricts individual freedom. From a Nordic perspective, this model makes meaningful freedom possible for more individuals in a given population (Hänninen et al., 2019). Social democracy is thus the governance that most effectively delivers upon the unintended teleology of the modern world: independence for individuals.

An interest in social democracy often comes entangled with conceptual confusion. When Bernie Sanders and Alexandria Ocasio-Cortez advocate “socialism,” they do not mean Stalinist or Maoist politics, but something similar to what the Nordics have (Washington Post, 2015; CBS News, 2019). This concept creep made the Oxford English Dictionary adjust its definition of “socialism” accordingly (OED, 2021). It is understandable that a majority of young Americans express that they would prefer this more egalitarian form of governance (Harris Poll, 2019). In the Nordic countries—Norway, Sweden, Denmark, Iceland, and Finland—income equality^1^, gender equality^2^, low-conflict politics, and prosperous economies with generous benefits contribute to high levels of happiness and social cohesion. Several United Nations rankings uphold these nations as facilitating the highest level of human well-being in the 21st century (Helliwell et al., 2020; UNDP, 2020a).

Since social democracy's golden age (1945–75), scholars and politicians have promoted the Nordic Model as suitable for universal export (Brandal et al., 2013). A scholarly argument has evolved that complicates this position (Sørensen and Stråth, 1997). Research reveals that the Nordic Model is undergirded by Lutheran norms and values (Stenius, 1997; Kildal and Kuhnle, 2005). The Protestant creed that was nationally embraced only in the Nordic region promotes strong work ethics, egalitarianism, togetherness, and civil duty. These values result in high labor force participation, but also motivate a willingness to cooperate closely at the national level, and to pay high taxes to ensure economic independence for a higher proportion of the population than what is the case in cultures with a Calvinist or Catholic heritage (Kahl, 2009).

During the Cold War, the Nordic Model was referred to as the Middle Way, meaning between liberalism and socialism. This term is misguided. The social-democratic model is predominantly liberal, viewing individuals—not groups—as the primary unit of governance (Harari, 2014). Catholic thinking drove an emphasis on uncovering natural law, and Calvinist thinking on granting abstract rights. In the Lutheran north, pragmatic rationalism motivated open-mindedness and experimentation (Gade Jensen, 2017). This ethos made Nordics more willing to deviate from political doctrine. When the Depression undermined the liberal ethos of *laissez faire*, these small countries adopted fascist corporatism to coordinate the national sphere to secure export competitiveness and minimize unemployment. Instead of trusting the invisible hand, employer and employee organizations commit to binding cooperation with the government. Together they rely on expert analysis to establish how much salaries can increase in a given year without hurting the export industry.

As a side effect, corporatism (or tripartism) provides Nordics with levers to restrain inequality. Instead of distributing growth solely through markets, tripartite collaboration can channel funds to workers with less market power, which feels appropriate in a culture influenced by Lutheran egalitarianism. This corporatist approach to salary negotiation results in a more compressed wage structure that makes people feel, to a greater extent, that everyone is in the same boat. For example, American medical doctors on average make over 8 times more than what grocery store workers do. In Norway, doctors make 2.5 times more^3^. Such relative income equality makes it more compelling to pool resources through high taxes, which can be viewed as socialist redistribution. These taxes fund fetus-to-funeral support, ensuring that no one falls below a certain level of economic comfort.

With free education and healthcare, high unemployment pay, and a line of other benefits, people are less dependent on each other. Historian (Trägårdh, 1997; Berggren and Trägårdh, 2012) coined the term *statist individualism* to describe the alliance between Nordic individuals and their rich, powerful state. High taxes empower the state to relieve individuals from burdensome social relations, furthering how the Henrichian coevolution freed Europeans from kin. Nordic parents rarely fund education, employers are easier to leave, and spouses are optional when even unemployed, single parents are secured a somewhat comfortable lifestyle. This independence could be cross-culturally salient, since it aligns with the ethos that marked the environment of our forager ancestors. Throughout humanity's agricultural phase, our ancestors were tied to intensive kinship practices, as these were necessary for building alliances that let them protect their fields. Henrich accounts for how such practices drove a psychology of conformity, dependence, and submission to authority, which was a break with our evolutionary past.

In the Paleolithic, our ancestors practiced extensive kinship, marrying into far-away bands to build expansive social networks that facilitated greater roaming range. For around 99.5% of the genus *Homo*'s history, human psychology evolved to facilitate a considerable level of independence. People formed temporary groups to solve distinct tasks, changed home base often, and developed a variety of social bonds suited to context. Moffett (2019) accounts for how, like only a handful of brainy mammals, humans became a fission-fusion species, meaning that we join and leave a variety of social groups throughout our lives. Unrelated nuclear families, often spanning three generations, fused for a period, separated, and later formed new constellations. Neolithic demands for social and spatial stability entailed a dramatic break. Only in the medieval and modern era could humans reembrace mobility, individualism, nuclear families, and flexible organization, which drove a psychology of non-conformity and independence (Henrich, 2020). In complex, modern societies, however, individuals can only acquire so much independence on their own.

Among urban dwellers, liberal economics lead to a Pareto distribution of independent winners at the top contrasted against miserable dependence at the bottom. For members of a species whose ancestors evolved in a forager ecology of reverse-dominance hierarchies (Bellah, 2011), it is not surprising that the grimness of this urban reality can make a majority agree that greater social spending would be beneficial. Still, research shows that the religious imaginaries that modern societies evolved from create national spectrums of “plausible policy options.” These cultural pasts can prevent a nation from uniting around policies that are more likely to provide the outcomes its people desire. Yale political scientist Sigrun Kahl substantiates that Western nations' religious pasts play a causal role in terms of cultural psychology, which strongly influences welfare preference. Such heritage expresses itself most clearly in how feeding the poor is viewed. Kahl writes that Catholic welfare (France, Italy, Spain) established that the poor have an unconditional right to be fed. Calvinist doctrine (Netherlands, England, United States) insisted that the able-bodied poor must be willing to work. Lutherans replaced this formal requirement with a strong social obligation to work. These differences arose in a distant past, but “affected the organizing principles of modern social assistance and the timing of its introduction” (Kahl, 2009, p. 268).

The Nordics' Lutheran past contributed to national imaginaries that, in the modern environment, make possible high productivity, effective cooperation, and widespread resource pooling. These outcomes facilitate an individual independence that aligns with our species' fission-fusion baseline. It is therefore understandable that other peoples are beguiled by the Nordic Model. Yet their cultural psychology may not lend itself to such governance: even if Nordic outcomes feel right, Nordic means may feel wrong. Becoming more aware of how our cultural imaginaries pre-consciously influence our political choices could empower populations to make decisions that are in their own interest—even if they experience instinctual resistance toward certain means. Raising such awareness has become pressing, as growing inequalities threaten social stability in many nations.

Fiction can help us examine our mental worlds in this regard. A culture's made-up stories illuminate which moral emotions underpin its citizens' political choices. In the Nordic region, Lutheran values are imbued in agonistic structure, that is, in how good vs. bad is embodied by protagonists and antagonists (Carroll, 2011; Carroll et al., 2012). Ingeborg Holm (1913) changed Swedish poverty legislation by crafting a thematic argument that aligns with Lutheran morals. The film's agonistic structure can be read as one between two Protestant creeds: good Lutheranism vs. bad Calvinism. American remakes of Scandinavian film exemplify how agonistic structure often must change when a story travels. I draw a line in terms of protagonist preference from fairy tales to film. Nordic noir crime fiction became a genre whose defining feature is to facilitate debate on how social democracy should adapt to counter environmental pressures. The genre's founding decalogy exemplifies how accessible fiction helps Nordics negotiate new adaptations for the relationship “between state, individual, and nation” (Nestingen, 2008, p. 256).

## A Silent Film's Lutheran Ideology

Martin Luther promoted that classes be united in a “priesthood of believers.” Such an egalitarian community was to be led by a king who, as head of a powerful state church, should secure every subject's salvation, but also their education and well-being. The state was meant to “guarantee the existence of a just society,” thus unifying spiritual and secular care. Everyone was responsible for contributing to a state within which all people, from king to beggar, are united by the “common good.” In Catholic societies, the Church was responsible for the poor. Their imaginary promoted that rich people give alms to ease their own way into heaven. The Lutheran safety net was a secular, local, and communal responsibility grounded in “neighborly love” (Lausten, 1995). To provide for those in need, the Lutheran Church, the rich, and regular people pooled resources in a “common fund,” which was the practical expression of poverty relief as a shared responsibility (Tønnessen, 2017).

This may sound heartwarming, but in the early modern era, the Nordic poor were no better off than if the region had remained Catholic (Lausten, 1995). Still, this imaginary of big government, religious egalitarianism, and civil duty found fertile ground. Black Death had killed more than half of the Scandinavian population in the 14th century, leading to a period with prehistoric levels of economic equality (Benedictow, 2016; Lagerås, 2016). Since the Late Iron Age, social stratification had been strong (Price, 2015). Between Black Death and the Reformation, Nordics enjoyed economic sameness as a result of agricultural land being widely available, while labor was expensive due to a shortage of people. In this environment, the Lutheran ethos felt compelling. Yet only when modern economic growth kicked in, could the region afford to fund its long-held preference for poor relief.

Ingeborg Holm (1913) offers insight into how a Lutheran past informs moral emotions. The film and the 1906 play it was adapted from closely align their thematic arguments with Lutheran sensibilities (Larsen, 2021). The film's eponymous protagonist is a middle-class mother whose entrepreneurial husband dies. When she needs temporary support, the local poverty board is unwilling to cover her basic expenses. Ingeborg's children are boarded out to the lowest-bidding caretakers, while she is institutionalized in a workhouse, permanently removing her from the labor force.

The director, Victor Sjöström, furthers the Lutheran argument of playwright Nils Krok. Like Luther, Krok emphasizes that begging is an evil that communities must eradicate by providing those unable to work with a share of society's resources commensurate to that of someone socially equal. “The needy can go to the poor relief without humiliating themselves,” Ingeborg's husband explains, “but walking from door to door is demeaning” (Krok, 2008, p. 31). Such an emphasis on the dignity of the individual is a core tenet of Lutheranism and social democracy; being independent of the whims of private charity is paramount.

To contrast these protagonistic values, the poverty board expresses Calvinist-aligned principles. The leader proudly proclaims that he has been able to reduce the poor tax every year, adding that “our system is a damned good system, because it is cheap” (p. 109). Such small-government attitudes, writes economist Robert Nelson, evokes Calvinism:

Where Lutheranism later became the dominant national religion, there was typically a state church headed by a Prince, King or other holder of state authority who oversaw what amounted to Lutheran theocracy. Calvinists, by contrast, typically made strong efforts to separate the institutional church of the Christian faithful from state control. (Nelson, 2017, p. 19)

These distinct attitudes toward resource pooling and ceding power to a central authority inform why the United States and the Nordic countries chose different welfare regimes. Lutheranism had accustomed Nordics to the unity of social care and state power. The institutional space being vacated by the Church could with relative ease be filled by social-democratic governance. People had had centuries of experience with what is referred to as a *maternalist* state, one that is well-meaning, tells people what to do, and provides for their needs (Brandal et al., 2013). Americans had prayed locally and felt greater distrust toward those state institutions that began to demand higher taxes.

Krok makes board members express Calvinist-aligned views on private charity, as well. Bengtsson sponsors coffee and snacks for the workhouse population in a way that villainizes him. The Lutheran social contract requires that people work diligently so that their taxes can make the state wealthy enough to support all citizens. Poverty care should be impersonal, a matter between individual and state. American Calvinism, writes theologian Henrietta Gronlund, took a form that “sharply differs from Nordic Lutheranism with regard to the relationship between government and the people, the responsibility or calling of the individual, the viewpoint toward business, and the role of philanthropy” (quoted in Nelson, 2017, p. 130). The American practice of listing donors in varying font size would mostly be untenable in a Lutheran-informed culture. Therefore, when Bengtsson wants to treat the poor on his own birthday, and insists that they be informed of who paid for the charity, this paints him as a moral outsider.

Krok also infuses board members with Calvinist prosperity gospel in terms of idealizing the rich and successful (p. 106). Luther was more skeptical of business ventures and wealthy people. His “employment ethic” contrasts the Calvinist “work ethic.” Instead of promoting hard work to succeed economically, Luther emphasized that employment itself is paramount, as any job can help people feel a sense of ordinariness, fulfillment, and moral satisfaction (McKowen, 2020). This ethos marks the discussion Ingeborg and her husband have when they invest their hard-earned money to open up a shop. That the play and film were so aligned with Lutheran values helped audiences realize that early 20th century poor care went against their cultural preference. In 1918, new laws were passed that prevented poverty boards from depriving poor people of civil rights. Old people were given their own institutions, and welfare payments were significantly increased (Hedling, 2000). This was an early manifestation of what later would evolve into social-democratic welfare.

## The Scandinavian Super Underdog

How cultural pasts play out in contemporary fiction can be studied, for instance, in remakes of Scandinavian film. Through my analysis of 15 originals and 18 American remakes, from the 1930s to the 2010s, I found a clear trend. You could suspect that after buying rights to remake often commercially proven film, Americans would be hesitant to change the story. Yet agonistic structure is often adapted to match different cultural heroics. We know from folklore studies how when “a story or a story motif . . . moves from one cultural environment to another . . . one of the most common changes is that characters are altered to align with the tradition-dominant characters of the new cultural area” (Tangherlini, 2013, p. 181). American filmmakers must suspect that in their cultural area, Nordic egalitarianism, togetherness, and sameness could be less appealing. In many of the remakes, protagonists are therefore made to be more capable, better looking, and/or more aggressive—thus more individualistically superior and less in need of communal cooperation.

Compared to their American counterparts, some of these Scandinavian protagonists are what I refer to as *super underdogs*, that is, particularly disadvantaged. The Scandinavian super underdog can be traced from fairy tales to film and reality TV. Espen Ashlad represents a common character type in fairy tales, but nowhere is he as warmly embraced as in Norway. He spends his life poking around in ashes, showing no indication of talent or drive, until a troll must be defeated or a princess won. Ashlad then saves the day, imparting that even the seemingly least capable can be valuable contributors to their community. His continued popularity attests to how modern Scandinavians, too, are drawn to heroes who embody egalitarian values.

Similar super underdogs can be found in an early-2000s wave of nine Norwegian films that featured men with mental disability (Dancus, 2006). In the same decade, a line of docu/reality TV enthralled Swedes and Norwegians by chronicling the lives of people with intellectual disability. Embraced by critics and audiences, these series impart that no challenge is too big for being overcome with honest effort and communal support. After mastering challenges at home and work, these super underdogs were sent sailing and mountain climbing, and later to ski across Greenland and through the Northwest Passage. Similar TV concepts have been tried elsewhere but without the astounding success that they achieved in Scandinavia. Such an embrace—and romanticization—of those low in the social hierarchy is integral to the region's cultural heroics. While the Western ideal was the honorable gentleman, Nordics made the modest peasant the hero of their stories (Trägårdh, 1997). This role makes the underdog perspective extra influential, as “the peasant continues to haunt Nordic political culture as the prototype of the Nordic citizen” (Stenius, 1997, p. 168). That Nordics are so drawn to agonistic structure that undermines individualistic brilliance—while promoting sameness and cooperation—reflects their Lutheran past, but this cultural preference could have deeper roots.

Since the Paleolithic, sparsely populated lands with poor soil and deadly winters seem to have instilled in Nordics the importance of both collaboration and individual ruggedness (Berggren and Trägårdh, 2015; Price, 2015). Even the most talented were prey to circumstance. The Nordics' turn to agriculture was belated, similar to how they were late to be drawn into European civilization. These factors seem to have let them retain more of the independent ethos that was paramount to our forager ancestors. In 1877, Vladimir Solovyov, a Russian philosopher, wrote that Scandinavians had, since they were Germanic barbarians, preserved “the principle of unconditional personal freedom and the supreme value of the individual^4^.” These preferences, he thought, explained why Nordics united around Lutheranism. “Only a Russian,” writes cultural critic Nina Witoszek, “immersed in a culture of serfdom and of the total stifling of the individual, could see the matter so sharply” (Witoszek, 2011, p. 60). Social democracy, we can surmise, found more fertile ground in the Nordic region—compared to everywhere else—partially because, in the modern world, pooling resources is the best way to ensure that as many individuals as possible can remain independent.

Social-democratic thought originated in Germany around 1860. Part of the labor movement opposed socialist authoritarianism by committing to “aims of promoting democracy and increasing personal freedom” (Brandal et al., 2013, p. 2). To an extent, they agreed with Marx in that liberal markets make it so that “individualism is illusory” (Lefebvre, 1968, p. 117). They disagreed on how to empower regular people. Liberal freedoms, Marx argued, reduce man “to the egoistic, independent individual” (Marx, 2012, p. 53). His opposition to religion informs how he opposes the modern sanctification of individualism: “The political democracy is Christian to the extent that it regards every individual as the sovereign” (Marx, 2012, p. 41–42). Marx advocated a radical transformation of ownership and other “social relations . . . to render possible a future reconstruction of the individual on new foundations” (Lefebvre, 1968, p. 7).

Social democrats did not oppose modern individualism; they sought to ameliorate the inequalities that arose alongside 19th-century industrialization. They did not use a religious vernacular to justify their position, yet modern scholarship makes clear how Lutheranism was their preconscious foundation (Kildal and Kuhnle, 2005). Building on hegemonic thought, social democrats were unwilling to deprive individuals of rights; a primary goal of economic empowerment was to increase personal freedoms. Early on, some thought the socialist utopia of group equity could be a long-term goal—achieved through taxes instead of revolution—but 20th-century social democrats moved on from this position. Their aspirations aligned with Luther's ideology, in that all should be guaranteed enough resources to maintain individual dignity and social equality. Although he was skeptical of how spiritually well-aligned rich businessmen were, Luther was not against economic inequity in and of itself (Nelson, 2017). When his ideology was embraced in the Nordic region, it resonated not primarily due to local class difference, which was relatively small, but because togetherness and resource pooling had long been important in this cold outpost of Europe.

Socialism still posed a threat as social democracy's golden age came to a close. After World War II, strong economic growth had facilitated that Nordics pool ever larger resources. The ideology that in the early modern era had manifested itself as local communities handing out wood and grain via “common funds” (Tønnessen, 2017) drove a socioeconomic experiment with an unclear endpoint. Swedish public spending had been 7 percent of GDP the year *Ingeborg Holm* premiered. By 1947, this had grown to 17%. In 1965, 25% of all Swedish kronor were distributed through the public purse, on course to a 72% peak in 1993 (IMF DataMapper, 2021). As strong growth was replaced by stagflation, and all the most obviously beneficial social projects had been implemented, Nordics had to reevaluate whether continuing to increase taxes would be the stairway to heaven as some proposed. This was a period of countercultural socialist fervor, which favored even more radical resource pooling. Nordics had to decide which of these two ideologies was right for them. This discussion was complicated by how their secularized Lutheranism drove a conformism that hindered open debate. Politics of consensus made for effective governance, but drove a sameness of thought that could engender political blind spots. Literary critic Hägg (2005) writes that in post-war Sweden one could discuss anything but politics. Again—like with *Ingeborg Holm*—popular fiction let Swedes test their moral emotions against contemporary realities.

## Nordic Noir as Discourse Medium

Made-up stories were a medium that facilitated updates to the Nordic imaginary in a less contentious manner. Literature thus became more politicized than what was common in countries with greater parliamentary disunity (Hauge, 2013). An influential example is *The Story of a Crime* (1965–75), the originating decalogy of what would later be termed Nordic noir. Maj Sjöwall and Per Wahlöö adopted the American police procedural, which had replaced the previous era's superior sleuths with cops who solve more realistic crime. The Marxist authors turned the conservative genre into a vehicle for far-left critique. They sought to convince working-class readers that social-democratic reform did not go far enough. Sjöwall explains

Wahlöö had written political books, but they'd only sold 300 copies. We realized that people read crime and through the stories we could show the reader that under the official image of welfare-state Sweden there was another layer of poverty, criminality and brutality. We wanted to show where Sweden was heading: toward a capitalistic, cold, and inhuman society (France, 2009).

The decalogy builds toward a Marxist critique that today can read as bizarre. Income equality had increased for decades, yet Sjöwall and Wahlöö dramatize a Sweden on the brink of becoming a hellscape of crime and exploitation. They held back on overt ideology until the popularity of the first novels had hooked readers. The decalogy's moral center is conveyed in the final novel, in which a welfare child murders the Prime Minister. This makes the killer “wiser and more right-thinking [than] most of us.” She sees more clearly “the corrupt rottenness of society . . . than thousands of other young people. As she lacks political contacts and has little idea of what is involved in a mixed-economy government, her clarity of vision is even greater” (Sjöwall and Wahl, 2007). The novel's final word is “Marx.” Only his ideology—the decalogy argues—can save Swedes from the evils of social democracy.

In spite of these ideological excesses, the series was embraced by readers across the political spectrum. Sjöwall and Wahlöö sold over 10 million books worldwide. Their prescription for cure was misguided, but their systemic analysis was perceived as incisive and relevant. Social Democrats had been in power since the 1930s, leading Swedes through an enormous transformation. But who knew the long-term consequence of handing out so much free money? Another fear was that American pop culture could dilute those beliefs that made Scandinavians altruistic. In Nordic noir, foreign and domestic threats inform crimes that are solved by police who embody Nordic values. Which beliefs to retain and which to leave behind was a crucial question throughout the post-war period. As modernization caught speed, Swedes even abandoned Lutheranism (Tomasson, 2002).

As the 1960s came to an end, the world had changed so much that simply finding new projects to channel tax revenue to no longer sufficed as a political strategy. In the post-war years, there had been remarkable unity behind public expansion. Political scientist Herbert Tingsten declared the end of politics, as party difference appeared mostly to restrict itself to how quickly new policies should be implemented (Hadenius, 2003). The year after Sjöwall and Wahlöö's series ended, Social Democrats were voted out of office. Over the next decades, Nordic crime fiction established itself as a format whose distinguishing feature was to identify contemporary threats and dramatize these through illuminating crimes. While *The Story of a Crime* ended by prescribing a Marxist solution, the custom became only to identify problems so that the general public could discuss how to counter them.

Such an inclusive process evokes how theologian Forell (1983) conceptualizes Luther's Reformation to be a movement whose tenets should be continuously debated within all social strata. If only elites discuss cultural adaptation, Lutheran togetherness would weaken. In this tradition, Nordic noir and other accessible fiction took on an important role in terms of influencing the region's cultural psychology. From around 1965, critic Andrew Nestingen identifies a “weakening of the avant-garde as a measure of national culture [and] increased use of popular forms for political purposes” (Nestingen, 2008, p. 12). As with most claims regarding fiction's influence, we cannot establish precisely how, or to what extent, Nordic noir helped steer the region. Still, Nestingen concludes that popular fiction contributed “to the definition of a new middle ground between state, individual, and nation” (p. 256).

## Idealizing Nordic Success

With neoliberal globalization, many a eulogy was sung for social democracy. As Social Democratic parties outside of the Nordic region lost influence, many experts believed it would only be a question of time before the Nordics, too, had to join Western normalcy (Geyer et al., 2000). Their high-tax model appeared uncompetitive in a global economy^5^. Yet as the new millennium un-folded, the Nordic Model shone brighter than ever. Effective debate, partially facilitated through fiction, had helped the Nordics course-correct. Taxes as a percentage of GDP were kept below 50 (OECD Data, 2020). A few hurdles were erected to prevent welfare abuse, as global pop culture was perceived to influence Nordic youth in a manner that threatened their “employment ethic” (Larsen, 2020). Marginal tax rates were reduced for high earners, and much neoliberal adaptation was meant to make the job-creator class feel more welcome. This was not a break with Lutheran or social-democratic ideology, but an adjustment; the Nordic Model primarily entails that the middle class accepts lower salaries and higher taxes, so that the lower class can be paid more and receive higher benefits.

As other parts of the world grew despondent after the Great Recession (Foa and Mounk, 2019), the Nordic region became a myth “of the liberal-left imagination, in which happy, smiling children are polite to each other as they grow up to be pacifist social democrats eager to pay more taxes” (Rentoul, 2006). The caricature is fitting. Three-quarters of Norwegians consider their high taxes to be at an appropriate or too low level (Opinion, 2017). Even a large majority of Conservative Party voters agree (Infact, 2017). Half of Americans think that their much lower taxes are too high (Gallup, 2018). At the same time, we can sense a growing cultural embrace of higher social spending being necessary also in America. Political philosopher Michael Sandel's *The Tyranny of Merit* (Sandel, 2020) argues for an ethos that aligns quite precisely with social democracy. The Harvard professor's emphasis on the dignity of the individual and ordinary work resonates with Luther's teachings.

Such scholarly arguments, in combination with the populist-left movement represented by Bernie Sanders and Alexandria Ocasio-Cortez, suggest that America could be ready for social democracy. That half of young Americans are on board implies the same. Yet there seems to be a misconception among many that the Nordics pay for welfare through taxing the top percentile. This is not the case. In a world where capital and corporations travel, while most workers do not, neither can Nordics overly tax the prosperous. Per capita, there are more rich people in the Nordic countries than in the U.S^6^. Americans and others appear enthusiastic about Nordic outcomes, but they would likely be more hesitant if they understood which means were employed to produce these results.

In American politics, a common view is that there is “no better way to kill an idea than to assert that enacting it will require “raising taxes on the middle class” (Bernstein, 2019). Biden won the 2020 election promising only to increase taxes for those making over $400,000 (Konish, 2021). Even Elizabeth Warren and Bernie Sanders insisted that their policies would have a net positive effect on middle-class wallets (Viser and Sullivan, 2019). Under a welfare regime like the Nordic, not only would the middle class have to be willing to consume less, but those with political disagreements would have to be able to agree. The corporatist model requires a cultural psychology that facilitates negotiation and consensus (Stenius, 1997). Before Nordics make significant changes, a long, inclusive process precedes decision. Experts can spend years preparing advice before all involved parties chime in. Disagreement may be considerable, but when a new course has been negotiated, political parties may agree to vote unanimously. This lessens the risk of refighting. “The negotiated or ‘corporatist' economy means that political decision-making often takes time,” writes Brandal and Thorsen, but the result is a high level of transparency, legitimacy, and trust (Brandal and Thorsen, 2018, p. 163).

Centuries of being indoctrinated into a Lutheran story of everyone belonging in the same “priesthood of believers” make this deliberative process more compelling to Nordics. Few countries have a cultural history that lends itself to such corporatism. Contemporary American politics suggest that consensus and close cooperation are not readily available tools for change. Without this element of fascist corporatism, socialist redistribution through taxes becomes more challenging. Even the very concept of tax triggers different moral emotions in the U.S. Instead of viewing taxes as resource pooling, they are often conceptualized as governmental greed. America was founded on tax revolt, and tax resistance has remained a theme ever since. Nordic outcomes may seem enviable compared to current American dysfunction, but the actual mechanisms of Scandinavian social democracy appear misaligned with American cultural psychology.

## Conclusion

Henrich's account of how the modern world resulted from the Church's dissolution of European tribes traces how Western psychology has coevolved with changing institutions. This process has entailed a return toward the independent ethos of our forager ancestors. I have argued that the Nordics' social-democratic adaptation to modernity produced the most functional political model. This is not an irresistible argument, but given the performance of Nordic countries in terms of productivity and populace satisfaction, it is a reasonable one. The region's norms and values at the beginning of the 20th century motivated these countries to organize their economic and social spheres in a manner that was rewarded by historical coincidence. Lutheran togetherness happened to make possible the comprehensive welfare regimes that were much needed in an industrialized environment. Not only did a generous safety net reduce human suffering and increase happiness, but it provided Nordic economies with several competitive advantages (see note 5).

Emulating Nordic success is made difficult, I argue, due to differences in cultural psychology, which is a product of historical influence. The rise of social democracy, writes Francis Fukuyama, “is full of historical accidents and contingent circumstances that cannot be duplicated” (Fukuyama, 2012, p. 434). Although the past cannot be changed, we could strive to free our psychologies from the prejudices that our past imposes on us. If I am correct in that the independence that social democracy facilitates could be cross-culturally salient, other nations could benefit from doing what the Nordics do—even if the particulars of such governance misalign with their cultural intuition. Naturally, in many parts of the world, upholding “independence for individuals” as a primary value would be frowned upon—particularly in those kinship societies that still exist. Yet the relative ease with which non-Western populations have adapted to what Henrich terms WEIRD practices attests to the appeal of a psychology evocative of foragers^7^. I argue not that our species stopped evolving after the Neolithic; that process accelerated (Cochran and Harpending, 2009). But in the 5,000 years since Nordics self-domesticated to cultivate fields (Price, 2015), the fission-fusion baseline of their psychology seems not to have changed that dramatically. Our species' remarkable flexibility allowed us to suppress our drive toward independence, but once intensive kinship practices were no longer advantageous, people were quick to abandon them. Fukuyama (2014) writes that these practices “among the Germanic barbarian tribes dissolved within a generation or two of their conversion to Christianity.”

If humans have retained an innate drive toward this evolutionary baseline, social-democratic outcome appears not anomalous, but predictable. Additional independence from parents, employers, and spouses allows a freer expression of our fission-fusion sociality. *Homo sapiens* being perennially torn between our impulse toward independence and our social and other needs informs what Immanuel Kant termed our “unsocial sociability” (Kant, 1970, p. 44). This social instability, the foundation for our fission-fusion psychology, makes Trägårdh conclude that social democracy is the most effective cultural adaptation to free markets (Berggren and Trägårdh, 2015, p. 388). Liberal ideology may posit that independence and individualism go hand in hand. On the American prairie, this may have been at least poetically true. But for urban dwellers, a significant proportion will not perform well enough in free markets to earn meaningful independence. Individuals can pull themselves up by their bootstraps, but the lowest quartile will remain the lowest quartile.

That social-democratic governance is better aligned with human nature is supported by the World Happiness Report (Helliwell et al., 2020). Every year of its existence, the WHR has ranked the five Nordic countries among the top 10 happiest nations. In 2017, 2018, and 2019, Nordic countries held the top three spots. If nations without a Lutheran heritage are serious about reducing inequalities and political conflict, the Nordics have lessons to offer. If social-democratic outcome appears sufficiently appealing, then how they cooperate and resource-pool can still be emulated—if non-Nordic populations can be convinced to act against what their moral emotions tell them to prefer.

To what extent this is possible, and which methods would best facilitate such a process of cultural deprogramming, are unclear. Greater awareness around how our religious heritage plays a causal role in our cultural psychology could be a step in the right direction. One tool for achieving this could be to show in fiction how our cultural prejudices inform agonistic structure. If those emotions for good and bad that were programmed into a population no longer facilitate the outcomes they desire, it could be reasonable to move past those moral emotions. Much Nordic fiction illustrates which Lutheran-aligned emotions underpin social-democratic success. Such insights should be taken to heart by those politicians and activists who argue for social democracy, but refer to it as “socialism.” Crucially, a better understanding of the Nordic Model could help them steer clear of actual socialism, which is underpinned by a very different morality. The difference in outcome between societies governed by social-democratic vs. socialist values can hardly be overemphasized.

## Author Contributions

The author confirms being the sole contributor of this work and has approved it for publication.

## Conflict of Interest

The author declares that the research was conducted in the absence of any commercial or financial relationships that could be construed as a potential conflict of interest.

## Publisher's Note

All claims expressed in this article are solely those of the authors and do not necessarily represent those of their affiliated organizations, or those of the publisher, the editors and the reviewers. Any product that may be evaluated in this article, or claim that may be made by its manufacturer, is not guaranteed or endorsed by the publisher.

## Footnotes

^1^The Nordic countries generally rank among the rich world's most income-equal nations. The United States is often ranked as the rich world's most income-unequal nation. See World Bank (2019), Central Intelligence Agency (2021) and UNDP (2020b).

^2^UNDP (2020c) ranks all Nordic countries among the top 10 most gender-equal nations. The United States ranks 46th.

^3^From Statistics Norway and U.S. Bureau of Labor Statistics, cited in Eia (2020).

^4^Solovyov (1966–69) quoted in (Witoszek, 2011, p. 60).

^5^High taxes are a burden, but the Nordic Model also generates a line of competitive advantages. A compressed wage structure makes high-skilled labor relatively cheaper, which pushes production up the value chain. Expensive low-skilled labor gives employers little choice but to invest more capital per worker, making innovation and automation key. This results in higher productivity per worker, which combined with high labor force participation grows each country's economic pie. Pooling resources helps educate the workforce, keeps workers healthier, lets entrepreneurs feel safe to take risk, and allows obsolete workers to acquire new skills. The result is well-coordinated nations that do well in between-nation competition, while providing within-nation security for all citizens.

^6^For a popular account, see Eia (2016); or read the transcript: http://evonomics.com/where-in-the-world-is-it-easiest-to-get-rich/.

^7^Henrich coined the acronym WEIRD to bring attention to how claims of psychological universals often build on research conducted exclusively on American undergraduates or other populations who are Western, Educated, Industrialized, Rich, and Democratic. See Henrich et al. (2010).

## References

[B1] Bellah R. N. (2011). Religion in Human Evolution: From the Paleolithic to the Axial Age. Cambridge, MA: Harvard University Press. 10.4159/harvard.9780674063099

[B2] Benedictow O. J. (2016). The Black Death and Later Plague Epidemics in the Scandinavian Countries: Perspectives and Controversies. Berlin: De Gruyter. 10.1515/9788376560472

[B3] Berggren H. Trägårdh L. (2012). Social trust and radical individualism: the paradox at the heart of nordic capitalism, in Nordic Way: Equality, Individuality and Social Trust (Stockholm: Swedish Institute), 13–29.

[B4] Berggren H. Trägårdh L. (2015). Är svensken människa? Gemenskap och oberoende i det moderna Sverige. Stockholm: Norstedts.

[B5] Bernstein J. (2019). We Can't Fund the Progressive Agenda by Taxing the 1% Alone. Vox. Available online at: https://www.vox.com/2019/8/2/20751074/middle-class-taxes-medicare-sanders-warren-debate (accessed August 2, 2019).

[B6] Brandal N. Bratberg Ø. Thorsen D. E. (2013). The Nordic Model of Social Democracy. New York, NY: Palgrave Macmillan. 10.1057/9781137013279

[B7] Brandal N. Thorsen D. E. (2018). Between individualism and communitarianism: the nordic way of doing politics, in Sustainable Modernity: The Nordic Model and Beyond, eds N. Witoszek and A. Midttun (New York, NY: Routledge), 160–186. 10.4324/9781315195964-9

[B8] Carroll J. (2011). Reading Human Nature: Literary Darwinism in Theory and Practice. Albany, NY: SUNY Press.

[B9] Carroll J. Gottschall J. Johnson J. A. Kruger D. J. (2012). Graphing Jane Austen: The Evolutionary Basis of Literary Meaning. New York, NY: Palgrave Macmillan. 10.1057/9781137002419

[B10] CBS News (2019). Alexandria Ocasio-Cortez: The ‘60 Minutes' interview. 60 Minutes. Available online at: https://www.cbsnews.com/video/alexandria-ocasio-cortez-congresswoman-challenging-democratic-establishment-60-minutes-interview-2019-06-23/ (accessed June 24, 2019).

[B11] Central Intelligence Agency (2021). The World Factjournal. Available online at: https://www.cia.gov/the-world-factjournal/field/gini-index-coefficient-distribution-of-family-income/country-comparison (accessed August 24, 2021).

[B12] Cochran G. Harpending H. (2009). The 10,000 Year Explosion: How Civilization Accelerated Human Evolution. New York, NY: Basic Journals.

[B13] Dancus A. M. (2006). Male mental illness and Norwegian compassion: a study of *Elling* (2001) and the art of negative thinking. Nord. J. Masc. Stud. 8, 7–25. 10.18261/ISSN1890-2146-2013-01-02

[B14] Eia H. (2016). Where in the World is it Easiest to Get Rich? TEDxOslo. Available online at: https://www.youtube.com/watch?v=A9UmdY0E8hU (accessed May 3, 2016).

[B15] Eia H. (2020). Sånn er Norge: Rik og lik. Oslo: NRK.

[B16] Foa R. S. Mounk Y. (2019). Youth and the populist wave. Philos. Soc. Crit. 45, 1013–1024. 10.1177/0191453719872314

[B17] Forell G. (1983). The Luther Legacy: An Introduction to Luther's Life and Thought for Today. Minneapolis: Augsburg Publishing House.

[B18] France L. (2009). The Queen of Crime. The Guardian. https://www.theguardian.com/journals/2009/nov/22/crime-thriller-maj-sjowall-sweden (accessed November 22, 2019).

[B19] Fukuyama F. (2012). The Origins of Political Order: From Prehuman Times to the French Revolution. New York, NY: Farrar, Strauss and Giroux.

[B20] Fukuyama F. (2014). Political Order and Political Decay. New York, NY: Farrar, Strauss and Giroux.

[B21] Gade Jensen H. (2017). Luther var ikke demokrat. Og mange tak for det. Politiken.

[B22] Gallup (2018). https://news.gallup.com/poll/232361/less-half-say-taxes-high.aspx (accessed April 16, 2018).

[B23] Geyer R. Ingebritsen C. Moses J. W. (eds) (2000). Globalization, Europeanization and the End of Scandinavian Social Democracy? New York, NY: St. Martin's Press. 10.1057/9780230371651

[B24] Hadenius S. (2003). Modern svensk politisk historia: konflikt och samförstånd. Stockholm: Hjalmarsson och Högberg.

[B25] Hägg G. (2005). Välfärdsåren: Svensk historia 1945–1986. Stockholm: W og W.

[B26] Hänninen S. Lehtelä K.-M. Saikkonen P. (2019). Introduction: the nordic welfare state as a state of civilization, in The Relational Nordic Welfare State: Between Utopia and Ideology, eds Hänninen, Lehtelä, and Saikkonen (Cheltenham: Edward Elgar Publishing), 1–14. 10.4337/9781788974653.00006

[B27] Harari Y. N. (2014). Sapiens: A Brief History of Humankind. New York, NY: Random House.

[B28] Harris Poll (2019). Percent of Millennials and Gen Z Agreeing with Statement. Available online at: https://www.axios.com/exclusive-poll-young-americans-embracing-socialism-b051907a-87a8-4f61-9e6e-0db75f7edc4a.html (accessed March 10, 2019).

[B29] Hauge H. (2013). Nordic sameness and difference, in Law and Justice in Literature, Film and Theater: Nordic Perspectives, ed K. M. Simonsen (Berlin: De Gruyter), 25–44.

[B30] Hedling E. (2000). Swedish cinema alters history: *Ingeborg Holm* and the poor laws debate. Scandinavica 39, 1, 47–64.

[B31] Helliwell J. F. Layard R. Sachs J. D. De Neve J.-E. (2020). World Happiness Report 2020. Available online at: https://worldhappiness.report/ (accessed March 31, 2021).

[B32] Henrich J. (2020). The WEIRDest People in the World: How the West Became Psychologically Peculiar and Particularly Prosperous. New York, NY: Farrar, Straus and Giroux.

[B33] Henrich J. Heine S. J. Norenzayan A. (2010). The weirdest people in the world? Behav. Brain Sci. 33, 61–83. 10.1017/S0140525X0999152X 20550733

[B34] IMF DataMapper (2021). Government Expenditure, % of GDP. Available online at: https://www.imf.org/external/datamapper/exp@FPP/SWE (accessed August 24, 2021).

[B35] Infact (2017). Verdens Gang. Available online at: https://www.vg.no/nyheter/innenriks/i/0agrJ/seks-av-ti-hoeyre-velgere-mener-skattenivaaet-er-passe-hoeyt (accessed March 9, 2017).

[B36] Ingeborg Holm (1913). Dir V. Sjöström. Sweden.

[B37] Kahl S. (2009). Religious doctrines and poor relief: a different causal pathway, in Religion, Class Coalitions, and Welfare States, eds K. van Kersbergen and P. Manow (Cambridge: Cambridge University Press), 267–295. 10.1017/CBO9780511626784.011

[B38] Kant I. (1970). Idea for a universal history with a cosmopolitan purpose, in Kant's Political Writings, ed H. S. Reiss, transl. by H. B. Nisbet (Cambridge: Cambridge University Press), 41–53.

[B39] Kildal N. Kuhnle S. (eds). (2005). Normative Foundations of the Welfare State: The Nordic Experience. London: Routledge.

[B40] Konish L. (2021). Biden has Promised Not to Raise Taxes on People Earning Less than $400,000. CNBC. Available online at: https://www.cnbc.com/2021/03/18/biden-tax-plan-what-people-making-under-and-over-400000-can-expect.html (accessed March 11, 2021).

[B41] Krok N. (2008). Ingeborg Holm. Umeå: Atrium.

[B42] Lagerås P. (ed). (2016). Environment, Society and the Black Death: An Interdisciplinary Approach to the Late-Medieval Crisis in Sweden. Oxford: Oxbow Journals. 10.2307/j.ctvh1dr32

[B43] Larsen M. (2020). Sealing new truths: film adaptation as cultural capstone for *101 Reykjav*í*k*. J. Scand. Cinema 10, 25–44. 10.1386/jsca_00012_1

[B44] Larsen M. (2021). Investigating the lutheran roots of social democracy in *Ingeborg Holm*. Scand. Stud. 93, 505–532.

[B45] Lausten M. S. (1995). The early reformation in Denmark and Norway 1520–1559, in The Scandinavian Reformation: From Evangelical Movement to Institutionalisation of Reform, ed Ole Peter Grell (Cambridge: Cambridge University Press), 12–41. 10.1017/CBO9780511758690.004

[B46] Lefebvre H. (1968). The Sociology of Marx. New York, NY: Pantheon Journals.

[B47] Marx K. (2012). Selected Essays. Auckland: Floating Press.

[B48] McKowen K. (2020). Substantive commitments: reconciling work ethics and the welfare state in Norway. Econ. Anthropol. 7, 120–133. 10.1002/sea2.12169

[B49] Moffett M. W. (2019). Human Swarm: How Our Societies Arise, Thrive, and Fall. New York, NY: Basic Journals.

[B50] Nelson R. (2017). Lutheranism and the Nordic Spirit of Social Democracy: A Different Protestant Ethic. Aarhus: Aarhus University Press. 10.2307/j.ctv62hgm7

[B51] Nestingen A. (2008). Crime and Fantasy in Scandinavia. Seattle: University of Washington Press.

[B52] OECD Data (2020). Revenue Statistics. Available online at:https://data.oecd.org/tax/tax-revenue.htm (accessed August 24, 2021).

[B53] OED (2021). Socialism. Oxford English Dictionary. Available online at: https://www.oed.com/view/Entry/183741 (accessed August 24, 2021).

[B54] Opinion (2017). Avisenes Nyhetsbyrå. Available online at: https://www.klartale.no/norge/folk-er-fornoyd-med-skatten-1.1053978 (accessed November 10, 2017).

[B55] Price T. D. (2015). Ancient Scandinavia: An Archaeological History from the First Humans to the Vikings. Oxford: Oxford University Press.

[B56] Rentoul J. (2006). Pay Attention, Rebels! Independent on Sunday. Available online at: https://www.independent.co.uk/voices/commentators/john-rentoul/john-rentoul-pay-attention-rebels-6110975.html (accessed August 24, 2021).

[B57] Sandel M. J. (2020). The Tyranny of Merit: What's Become of the Common Good? New York, NY: Farrar, Straus and Giroux.

[B58] Sjöwall M. Wahl,öö P. (2007). Terrorists [Terroristerna]. Transl. by J. T. Blair. London: Harper Perennial.

[B59] Solovyov V. (1966–69). Sobranjie socinienji W. S. Solovjova, Vol. I. Brussels.

[B60] Sørensen Ø. Stråth B. (1997). The Cultural Construction of Norden. Oslo: Scandinavian University Press.

[B61] Stenius H. (1997). The good life is a life of conformity: the impact of lutheran tradition on nordic political culture, in The Cultural Construction of Norden, eds Ø. Sørensen and B. Stråth (Oslo: Scandinavian University Press), 161–171.

[B62] Tangherlini T. R. (2013). Danish Folktales, Legends, and Other Stories: The Danish Folklore Nexus. Seattle: University of Washington Press.

[B63] Tomasson R. F. (2002). How Sweden became so secular. Scand. Stud. 74, 61–88.

[B64] Tønnessen A. V. (2017). Reformasjonen og utviklingen av en ny fattighjelp, in Reformasjonen i nytt lys, eds T. Rasmussen and O. Tjørhom (Oslo: Cappelen Damm Akademisk), 193–212.

[B65] Trägårdh L. (1997). Statist individualism: on the culturality of the nordic welfare state, in The Cultural Construction of Norden, eds Ø. Sørensen and B. Stråth (Oslo: Scandinavian University Press), 253–285.

[B66] UNDP (2020a). Human Development Reports. New York, NY: United Nations Development Programme. 1990–2019.

[B67] UNDP (2020b). Inequality-adjusted Human Development Index. Available online at: http://hdr.undp.org/en/content/inequality-adjusted-human-development-index-ihdi (accessed August 24, 2021).

[B68] UNDP (2020c). Gender Inequality Index. Available online at: http://hdr.undp.org/en/content/gender-inequality-index-gii (accessed August 24, 2021).

[B69] Viser M. Sullivan S. (2019). Will Medicare-for-All Hurt the Middle Class? Washington Post. Available online at: https://www.washingtonpost.com/politics/will-medicare-for-all-hurt-the-middle-class-elizabeth-warren-and-bernie-sanders-struggle-with-questions-about-its-impact/2019/10/05/a645fb3e-dbdb-11e9-ac63-3016711543fe_story.html (accessed October 5, 2019).

[B70] Washington Post (2015). The CNN Democratic Debate Transcript, Annotated. Available online at: https://www.washingtonpost.com/news/the-fix/wp/2015/10/13/the-oct-13-democratic-debate-who-said-what-and-what-it-means/ (accessed October 13, 2015).

[B71] Witoszek W. (2011). The Origins of the “Regime of Goodness”: Remapping the Cultural History of Norway. Oslo: Universitetsforlaget.

[B72] World Bank (2019). Gini Index. Available online at: https://data.worldbank.org/indicator/SI.POV.GINI/ (accessed August 24, 2021).

